# A Review of Data of Findings on Night Shift Work and the Development of DM and CVD Events: a Synthesis of the Proposed Molecular Mechanisms

**DOI:** 10.1007/s11892-018-1102-5

**Published:** 2018-10-20

**Authors:** S. Strohmaier, E. E. Devore, Y. Zhang, E. S. Schernhammer

**Affiliations:** 1 Department of Epidemiology, Center for Public Health, Medical University of Vienna, Kinderspitalgasse 15, 1090 Vienna, Austria; 2 Channing Division of Network Medicine, Harvard Medical School, Boston, MA USA; 3 Department of Epidemiology, Harvard T.H. Chan School of Public Health, Boston, MA USA

**Keywords:** Night work, Cardiovascular, Cardiometabolic, Diabetes, Circadian misalignment, Inflammation

## Abstract

**Purpose of Review:**

Night shift work has become highly prevalent in our 24/7 societies, with up to 18% of the US work force working alternate shift schedules. However, studies indicate that there may be adverse health effects of chronic night work across diverse populations. These effects are likely due to misalignment of the circadian system with work schedules, mediated by the system’s primary marker melatonin as well as other downstream molecules.

**Recent Findings:**

Melatonin has multiple biologic actions that are relevant to cardiometabolic disease, including modulation of oxidative stress, inflammation, and (via the melatonin receptor) vasoconstriction. Behavioral traits, such as chronotype and meal timing, have recently been shown to interact with the effects of night work on cardiometabolic health.

**Summary:**

Together with recent findings suggesting a role for circadian genes in cardiometabolic risk, the interactions of night shift work and behavioral traits are likely to facilitate novel treatment and prevention approaches for cardiovascular disease and type 2 diabetes, incorporating aspects of clock and timing.

## Introduction

Involvement of the circadian system in the pathogenesis of cardiovascular disease (CVD) has been suspected since older anecdotal evidence indicated a higher frequency of myocardial infarction in the early morning hours. More recent reports have continued to support this notion [1]. In the last couple of years, increasing attention has also been paid to the adverse health effects of night shift work (an important modulator of circadian rhythms) as they pertain to cardiometabolic health [2, 3]. In this review, we will summarize key epidemiological studies regarding the association of night shift work with type 2 diabetes (T2DM) and cardiovascular diseases risk, and provide a brief overview of the putative molecular mechanisms involved in these associations.

### Epidemiological Evidence

#### Epidemiological Studies of Night Shift Work and Type 2 Diabetes Risk

A number of epidemiologic studies have explored the association between shift work and diabetes in recent decades. The majority of these studies were conducted since 2000 across the United States (US), Europe, and Asia. A recent meta-analysis identified 12 such studies involving more than 200,000 participants, and reported that, overall, shift work history was related to an increased risk of diabetes [3]. Although the effect was statistically significant, it was modest as the odds ratio (OR) indicated a 9% greater diabetes risk (OR = 1.09, 95% confidence interval [CI] = 1.05–1.12) for those with any history of shift work compared to those without such a history. In addition, although the majority of participants in these studies were women, the observed association appeared to be stronger for men (OR = 1.37; 95% CI = 1.20–1.56) compared to women (OR = 1.09; 95% CI = 1.04–1.14). Associations were also stronger for rotating shift work compared to other types of shift work (irregular shifts, night shifts, mixed shifts, and evening shifts). Subgroup analyses indicated that results were not different according to study design, sample size, study location, or type of occupation [3].

A landmark study of rotating night shift work and diabetes (included in the above meta-analysis) was conducted by our group within the Nurses’ Health Study (NHS) and Nurses’ Health Study II (NHS II) in 2011 [4]. This study focused on more than 190,000 female nurses aged 25–67 at study baseline who reported their total number of years working rotating night shifts (defined as working ≥ 3 nights per month in addition to day and evening shifts in that same month) and their type 2 diabetes status (confirmed by a validated supplemental questionnaire) during 18–20 years of follow-up. We found a highly significant linear trend between increasing duration of rotating shift work history and greater diabetes risk (*P*_trend_ < 0.001). Specifically, compared to participants without such a history, participants with 1–2, 3–9, 10–19, and ≥ 20 years of rotating night shift work history had the following elevated risks of diabetes after adjustment for important confounding factors: hazard ratios (HRs) 1.05 (95% CI = 1.00–1.11), 1.20 (1.14–1.26), 1.40 (1.30–1.51), and 1.58 (1.43–1.74), respectively. These results were consistent across the two cohorts. Additional analyses adjusting for body mass index attenuated these estimates, although the overall trend remained highly significant; still, this result suggests that body weight might mediate the observed association.

In the younger cohort of Nurses’ Health Study II, a more recent analysis in 2015 indicated an interaction between rotating night shift work and diurnal preference (i.e., chronotype) in relation to diabetes risk [5**•**]. In particular, a mismatch in sleep and work timing appeared to increase the risk of diabetes slightly among early chronotypes who worked ≥ 10 years of rotating night shifts, and to increase the risk strongly among late chronotypes who worked no rotating night shifts. This finding is consistent with recent evidence suggesting a role of meal timing [6] and fasting time in the risk of type 2 diabetes (including postprandial glucose response [7]) as well as potentially also CVD [8].

Another key study was conducted in the Black Women’s Health Study cohort [9]. In this study, women aged 21–69 years old self-reported their history of night shift work (defined as the graveyard shift, midnight to 8 AM) and their diabetes status over 8 years of follow-up. Results indicated a highly significant trend of greater diabetes risk with increasing duration of night shift work (*P*_trend_ < 0.001). Compared to participants without a history of night shift work, those with 1–2, 3–9, and ≥ 10 years of night shift work had increased risks of diabetes: 1.14 (95% CI = 1.01–1.28), 1.18 (95% CI = 1.02–1.36), and 1.35 (95% CI = 1.13–1.62), respectively. Although the interaction of night shift work and obesity in relation to diabetes did not reach statistical significance (*p* = 0.12), stratified analyses suggested that the observed association was slightly stronger in obese women. This was the first study to extend previous findings of an association between shift work and diabetes to Black populations.

Overall, the current epidemiologic literature provides compelling evidence that night shift work may adversely affect metabolic health, particularly the risk of type 2 diabetes.

#### Epidemiological Studies of Night Shift Work and Cardiovascular Disease Risk

Epidemiological studies of shift work and cardiovascular disease (CVD) also have accumulated over the past few decades. These studies were largely conducted in Europe and the US, with sparse data coming from Asia and the Middle East. A systematic review and meta-analysis evaluated this literature in 2017. It evaluated 21 epidemiologic studies published between 2006 and 2016 with a total of 173,010 participants. The authors reported that, compared to non-shift workers, shift workers had a 17% increased risk of all CVD events, and an almost 20% increased risk of cardiovascular disease mortality.

Key studies were conducted in the NHS and NHS II with 22–24 years of follow-up [10**••**, 11]. One study found that rotating night shift work was significantly associated with an increase in risk of coronary heart disease (CHD). In NHS, compared to women without a history of shift work, women with < 5, 5–9, and ≥ 10 years of shift work history had CHD risks of 1.02 (95% CI = 0.97–1.08), 1.12 (95%CI = 1.02–1.22), and 1.18 (95%CI = 1.10–1.26), respectively, after adjustment for multiple possible confounding factors. In NHS II, the corresponding estimates were very similar across the same shift work categories. These associations persisted among women without any diagnosis of hypertension, hypercholesterolemia, or diabetes, although they were more prominent among obese women. There was also a suggestion that increased CHD risks observed in shift workers began to decrease after quitting shift work. A second study in NHS reported that compared with nurses who never worked night shifts, nurses with ≥ 5 years of rotating night shift work had significantly increased risk of CVD-related mortality. For nurses with 1–5, 6–14, and ≥ 15 years of rotating night shift work, their HRs for CVD mortality were 1.02 (95% CI = 0.94–1.11), 1.19 (95% CI = 1.07–1.33), and 1.23 (95% CI = 1.09–1.38), respectively.

In other countries, notable studies [12–14] have evaluated important associations between shift work and risk of CHD:

- In Finland, a study of approximately 1800 industrial male employees reported that shift workers had a relative risk (RR) of CHD of 1.27 (95% CI = 1.01–1.60) compared to day workers across 13 years of follow-up [14]. Additional analyses indicated an even higher risk of CHD among shift workers with adverse lifestyle factors (e.g., smoking and obesity) [13] and occupational factors (e.g., higher physical workload) [14].

- In Japan, a study of 17,600 workers over 23–25 years indicated that, compared with the day workers, rotating shift workers had a significantly higher risk of dying from ischemic heart disease (IHD) (RR 2.32, 95% CI = 1.37–3.95), whereas fixed-night work did not appear to be strongly associated with IHD (RR 1.23, 95% CI = 0.49–3.10) [12]. These associations were more pronounced in participants with additional coronary risk factors, such as smoking, hypertension, overweight, and habitual alcohol consumption, although tests for interactions did not reach statistical significance.

Taken together, epidemiological studies suggest associations of night shift work history with greater risk of CVD-related events, as well as possible subgroups of shift workers at particularly high risk. Elucidation of possible mechanisms underlying these associations is a key next step to facilitate interventions aimed at prevention of adverse cardiometabolic consequences.

### Mechanisms

#### Mechanisms for CVD

Atherosclerosis is a chronic disease characterized by abnormal localization of inflammatory cells and lipids in the sub-endothelial space [15–17] including insulin resistance, inflammatory cytokines (produced by adipocytes and the liver), and dysfunction of the endothelium. Specifically, cell adhesion molecules (e.g., E-selectin and intercellular adhesion molecule-1 [ICAM-1]) are expressed and recruit inflammatory cells, vascular smooth muscle cells are activated and cause vasoconstriction, and thrombogenesis is promoted via production of pro-thrombotic mediators [18].

#### Mechanisms for T2DM

Insulin resistance is a well-established pathogenic factor for T2DM [19, 20]. The vascular endothelium has been hypothesized to be a central site of insulin resistance in the insulin resistance syndrome [21, 22]. This unifying hypothesis localizes insulin resistance to the same tissue involved in the development of atherosclerotic CVD. As noted above, CVD itself is now understood to be, in part, a result of chronic subclinical systemic inflammation [23], giving rise to the hypothesis that insulin resistance and T2DM are, at least partly, inflammatory disorders [24–26].

#### The Circadian System

The word “circadian” is derived from Latin (“circa,” meaning roughly, and “dies” meaning day). The circadian system refers to the endogenous timing system that synchronizes physiology and behavior within 24-h day and night cycles, and enables anticipating and adapting to daily environmental changes [27]. The primary circadian pacemaker of this system in humans and other mammals is located in the suprachiasmatic nuclei (SCN), a region of the hypothalamus that coordinates molecular rhythms in all organs and cells throughout the body. In addition, peripheral oscillators are located in nearly every human tissue, including the heart, vessel walls, and β-cells of the pancreas.

Four families of core clock genes (Clock, Bmal1, Period [Per), Cryptochrome [Cry)] have been identified in all nucleated cells [28–30] that form interlocking positive and negative transcription-translation-based feedback loops. The net result is that gene products ultimately repress their own transcription in a delayed fashion, with negative feedback loops oscillating on approximately 24-h cycles. However, circadian genes and proteins do not cycle in a vacuum, as they also regulate so-called “clock-controlled genes” that are themselves canonical parts of the cycle. Clock-controlled genes are numerous; about 5 to 15% of the transcriptome is controlled by the core circadian genes and thereby follow a 24-h oscillation [31].

The circadian system is controlled by the “master pacemaker,” which regulates the sleep/wake cycle and pineal melatonin biosynthesis [32]. It also triggers the pineal gland to secrete melatonin (5-methoxytryptamine, the primary marker of the circadian system) during the dark phase of the light-dark cycle [33]. Direct light exposure during the night, which is perceived by specialized retinal photoreceptors that project monosynaptically to the SCN and pineal gland [34], suppresses melatonin production within 5 to 10 min of light perception, and this suppression persists until light is turned off, at which time melatonin production resumes.

Behaviors that induce misalignment between internal clock and external timing (e.g., night shift work, or traveling across time zones) have the potential to upset the inherent, roughly regular circadian rhythm in humans. Several studies have demonstrated negative effects of circadian misalignment on measurable biomarkers, including melatonin. Levels of melatonin’s major urinary metabolite, 6-sulfatoxymelatonin (aMT6s), are closely correlated with nightly peak plasma melatonin levels in blood and saliva, and therefore allow determination of nightly peak levels of melatonin in human studies [35]. In addition to aging, night shift work (with exposure to light at unnatural times of the day) has been associated with lower urinary aMT6s concentrations in numerous studies [36–39].

#### Mechanisms Linking Night Shift Work to T2DM and CVD Risk

Human observational studies have shown higher melatonin levels to be inversely associated with higher melatonin levels to be inversity associated with hypertesion, T2DM and incident myocardial infarction (MI). For example, in prospective, nested case-control studies of the NHS, after adjusting for an extensive set of confounding variables, the odds ratio for a unit decrease in log-transformed sulfatoxymelatonin:creatinine ratio was 1.48 (95% CI = 1.11–1.98) for T2DM and 1.40 (95% CI = 1.02–1.93) for incident MI [40, 41**•**]). Additional evidence for the protective effect of circulating melatonin on CVD/T2DM risk comes mostly from cross-sectional studies reporting decreased melatonin levels (which are also typically seen in night shift works) in patients with versus without diabetes [42, 43], and patients with versus without cardiovascular disease [44, 45].

Several lines of evidence from experimental studies support that melatonin may have a beneficial effect on glucose metabolism [42] and cardiovascular diseases [46, 47]. For example, melatonin has the ability to directly neutralize reactive oxygen and nitrogen species [48, 49], and stimulation of antioxidant enzymes involves the melatonin receptors MT1 and MT2 [50]. Reduction in endothelial oxidative stress has demonstrated benefits for endothelial function [51]. Moreover, by reducing reactive oxygen species, melatonin also suppresses NFκB which is the key transcription factor for inflammatory cytokines such as IL-6 and TNFα [52, 53]. In addition to these oxidative stress-related effects, animal data show that melatonin exerts influence on insulin secretion [54, 55], guards against β-cell damage [55, 56], and offsets metabolic disturbances (e.g., those induced by diabetes) [57–59]. Furthermore, cross-sectional human studies show that IL-6 and CRP levels follow light-dark patterns and are inversely related to melatonin [60–62], suggesting that melatonin enhances endothelial function and reduces inflammation [63, 64]. Indeed, melatonin receptors are found on endothelial cells [65], and melatonin affects vessel tension thereby increasing nitric oxide production [65–67]; this effect has also been demonstrated in humans when receiving endogenous melatonin [68, 69]. Melatonin receptors have been found on pancreatic islet β-cells, indicating the ability of melatonin to control insulin secretion as well [55], with adverse effects for night shift workers due to their lower nightly melatonin production.

Several associations between circadian gene variants and health outcomes have previously been described (e.g., sleep disorders [6] and obesity [70]). Recent studies, including genome-wide association studies (GWAS), have indicated significant associations between SNPs in *MTNR1B* (type 2 melatonin receptor) and T2DM, with odds ratios ranging from 1.09 to 1.20. Such findings provide further support for an association between the circadian system—and hence night shift work, which adversely affects this system—and cardiometabolic health [71–73]. Additionally, a variant in the *CRY2* gene has been associated with increased insulin resistance and T2DM at genome-wide levels of significance [74].

In summary, several lines of evidence, both from mechanistic studies as well as observational data in humans, provide support for a causal association between circadian disruption as encountered by night shift workers, and the primary circadian marker melatonin, with risk of T2DM and CVD. While night workers have been found to experience a higher risk of cardiometabolic diseases including T2DM and CVD, the mechanistic studies suggest that these effects are mediated by alterations in melatonin levels and melatonin’s function on melatonin receptors throughout the body, as well as through clock-genetic variants, and inflammatory and endothelial pathways common to CVD and T2DM.

## Conclusion

There is ample evidence for a variety of important pathways that are, at least partially, under clock control and suggest close involvement of the circadian system in cardiometabolic pathophysiology. Recent studies of behavioral traits, such as chronotype and corresponding meal timing, also suggest an interaction with night shift work [5], and future preventive efforts for T2DM and cardiovascular disease will likely incorporate such aspects of the biologic clock through chronotherapy or timed activities such as eating. With several important studies currently underway, we expect that our understanding of the interplay between the circadian system and cardiometabolic pathophysiology, and their utility in primary and secondary prevention, will continue to increase in the next few years.
